# Supplementary material for: Association between oral microbiome and seven types of cancers in East Asian population: a two-sample Mendelian randomization analysis
Source: Front Mol Biosci. 2023 Nov 21;10:1327893. doi: 10.3389/fmolb.2023.1327893 (PMC10702768; doi:10.3389/fmolb.2023.1327893)
Supplement: Supplementary file 1 [file DataSheet1.ZIP › Supplementary Materials/MR plots of saliva/saliva═╝/ovarian cancer/pheno.112_to_ovarian cancer_leave_one_out.pdf]

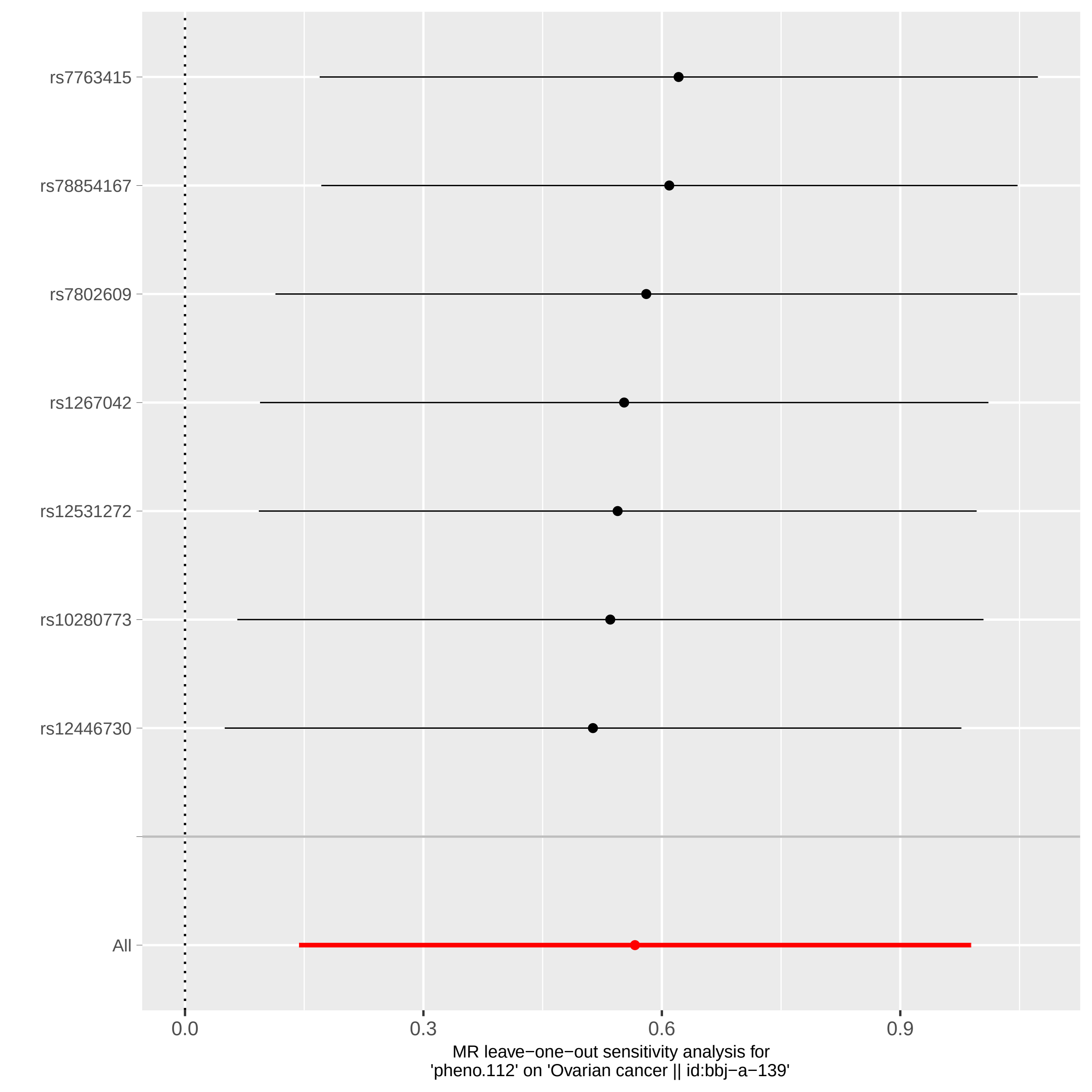

rs7763415
rs78854167
rs7802609
rs1267042
rs12531272
rs10280773
rs12446730
All
0.0
0.3
0.6
0.9
MR leave−one−out sensitivity analysis for
'pheno.112' on 'Ovarian cancer || id:bbj−a−139'
